# Transactions of the Medical Society of the State of New York for the Year 1891

**Published:** 1891-11

**Authors:** 


Transactions of the Medical Society of the State New York for the Year 1891. Edited by Frederic C. Curtis, M. D., Secretary of the Society. Octavo, pp. viii.—560. Published by the Society. 1891.

This volume of 560 octavo pages presents the work of this Society at its last meeting, and is most admirably edited by its accomplished secretary. The contents includes fifty-four scientific communications, besides reports of committees, obituary notices, lists of members and delegates, as well as members of the county medical societies, and, altogether, it is a most useful book, whether looked upon as a work of reference, or whether judged from its scientific excellence. There are 4,342 members of the county societies, and 919 members of incorporated voluntary medical societies, in affiliation with the State society. It has a representation of 147 delegates, and a permanent membership which aggregates 265, besides honorary and non-resident members.

In the volume before us there is so much of scientific interest, that it is difficult to particularize, but two subjects are of especial value, and ought to be read by every physician in the State. We refer to the discussions on Tuberculosis and Appendicitis. The former includes titles by Drs. H. R. Hopkins, Heneage Gibbs, John O. Roe, Samuel B. Ward, and E. L. Shurley, and these are followed by papers on the use of Koch's lymph, by Drs. Jacobi and Heineman. The subject of appendicitis is treated by Drs. Herman Mynter, Chas. McBurney, W. W. Keen, Lewis A. Stimpson, Geo. Ryerson Fowler, Robert F. Weir, and A. Vander Veer.

Another discussion that deserves notice is that on Pelvic Inflammation in Women, which was participated in by Drs. Andrew F. Currier, A. J. C. Skene, and Lewis S. McMurtry. Other papers of importance were presented by Dr. Jas. F. W. Ross, on An Inquiry into our Present Knowledge in the Progress of Myomatous Tumors; one on the Treatment of Gall-stones, by Dr. Wm. Wotkins Seymour; and one on Operative Procedures in Acute General Suppurative Peritonitis, by Dr. W. E. B. Davis, of Birmingham, Ala. The scientific value of this volume has not been excelled by any society proceedings with which we are familiar. Those members of the profession throughout the State who have not already obtained it, should not be slow to purchase. It is doubtful whether as much valuable literature can be obtained anywhere else for the price, namely, $1.50.

